# Comparative Assessment of In Vitro Xanthine Oxidase and α-Glucosidase Inhibitory Activities of Cultured Cambial Meristematic Cells, Adventitious Roots, and Field-Cultivated Ginseng

**DOI:** 10.3390/nu16030443

**Published:** 2024-02-02

**Authors:** Tianhe Zhang, Lijun Liu, Qiqi Chen, Yifei Wang, Xiujun Gao, Xingyi Ma, Peisheng Yan

**Affiliations:** 1 School of Marine Science and Technology, Harbin Institute of Technology, Weihai 264209, China; 22s030089@stu.hit.edu.cn (T.Z.); 19b929072@stu.hit.edu.cn (Q.C.);; 2 School of Science, Harbin Institute of Technology, Shenzhen 518055, China; 3 Biosen International, Jinan 250117, China; 4 Briteley Institute of Life Sciences, Yantai 264003, China; 5 Shandong Key Laboratory of Biochemical Analysis, College of Chemistry and Molecular Engineering, Qingdao University of Science and Technology, Qingdao 266042, China

**Keywords:** cambial meristematic cells (CMC), adventitious ginseng roots (AGR), field-cultivated ginseng roots (CGR), xanthine oxidase (XO), α-glucosidase

## Abstract

*Panax ginseng*, a traditional Chinese medicine with a history spanning thousands of years, faces overexploitation and challenges related to extended growth periods. Tissue-cultured adventitious roots and stem cells are alternatives to wild and field-cultivated ginseng. In this study, we assessed the in vitro xanthine oxidase and α-glucosidase inhibitory activities of saponin extracts among cultured cambial meristematic cells (CMC), adventitious ginseng roots (AGR), and field-cultivated ginseng roots (CGR). The xanthine oxidase (XO) and α-glucosidase inhibitory activities were determined by uric acid estimation and the *p*-NPG method, respectively. Spectrophotometry and the Folin–Ciocalteu, aluminum nitrate, and Bradford methods were employed to ascertain the total saponins and phenolic, flavonoid, and protein contents. The calculated IC_50_ values for total saponin extracts against XO and α-glucosidase were 0.665, 0.844, and >1.6 mg/mL and 0.332, 0.745, and 0.042 mg/mL for AGR, CMC, CGR, respectively. Comparing the total saponin, crude protein, and total phenolic contents revealed that AGR > CMC > CGR. To the best of our knowledge, this study presents the first report on the in vitro comparison of xanthine oxidase and α-glucosidase inhibitory activities among AGR, CMC, and CGR. The findings offer valuable insights into the development of hypoglycemic and antihyperuricemic medicinal, nutraceutical, and functional products utilizing AGR and CMC.

## 1. Introduction

Chronic metabolic diseases, including diabetes, hyperuricemia, and gout, are on the rise, primarily attributed to an irregular diet, extravagant nutrient intake, heightened mental stress, and a lack of physical exercise [1]. Approximately 537 million people were affected by diabetes in 2021, and projections indicate a further increase to 643 million by 2030 [2]. Lifestyle modifications and aging populations contribute to a global surge in hyperuricemia and gout cases. The prevalence of hyperuricemia varies, ranging from 11.3% to 47% in the United States of America, 11.9% to 25.0% in Europe, and 13.1% to 13.3% in the People’s Republic of China [3]. Recent studies have identified a correlation between hyperuricemia and an increased risk of developing diabetes [4,5,6,7]. Diabetes exhibits high prevalence among patients with hyperuricemia across all continents [8]. Diabetes and hyperuricemia are significant health concerns worldwide, with their occurrence and progression holding considerable relevance worldwide [9]. A confirmed positive association between diabetes mellitus and hyperuricemia has been demonstrated. One plausible explanation for this correlation is that insulin resistance serves as a shared pathophysiological mechanism underlying both diabetes and hyperuricemia [3]. In diabetes, hyperglycemia initially arises from the failure of cells to fully respond to insulin, a condition referred to as insulin resistance [10]. In the presence of insulin resistance, hyperinsulinemia may reduce the renal excretion of uric acid, thereby contributing to the increased occurrence and development of hyperuricemia [4]. Meta-analysis revealed a notable occurrence of diabetes in patients with hyperuricemia and gout. The study underscores the importance of controlling both blood glucose and uric acid levels in patients with hyperuricemia and gout, emphasizing their critical role in prevention of diabetes [7].

Xanthine oxidase (XO) plays a crucial role in uric acid production, converting hypoxanthine into xanthine and subsequently transforming xanthine into uric acid. This process contributes to the development of hyperuricemia and gout [11]. Therefore, XO is regarded as a potential target for managing hyperuricemia as well as gout. Currently, allopurinol and febuxostat, both xanthine oxidase inhibitors, are employed in clinical settings to lower uric acid levels. However, owing to associated side effects such as skin rashes, systemic vasculitis, gastrointestinal, hepatic, and renal toxicity [12], coupled with an increasing hyperuricemia-suffering population, there is a growing demand for novel natural substances with better inhibitory activity against xanthine oxidase and fewer side effects compared to allopurinol and febuxostat. Quy and Xuan reported that the ethyl acetate extract from *Cordyceps militaris* exhibited the most potent xanthine oxidase inhibitory activity when compared to extracts from hexane, chloroform, or aqueous residue. Further, the CM8 fraction obtained from the ethyl acetate extract through column chromatography exhibited the highest xanthine oxidase inhibitory activity, displaying the lowest IC_50_ value of 62.82 µg/mL. These results suggest that *Cordyceps militaris* may be beneficial for treating hyperuricemia [13].

Type 2 diabetes mellitus (T2DM or T2D) represents the most common form of diabetes, accounting for approximately 90% of all diabetes cases worldwide [2]. T2D is characterized by elevated fasting and postprandial blood glucose levels. In managing postprandial hyperglycemia, one effective approach involves inhibiting enzymes responsible for carbohydrate breakdown [14,15,16].

The enzyme α-glucosidase plays an important role in regulating blood glucose levels. In the small intestine, disaccharides like maltose and sucrose, derived from dietary carbohydrates, are broken down by α-amylase. Subsequently, α-glucosidase further degrades them into monosaccharides (for example, glucose) before absorption [17,18]. Inhibition of α-glycosidase activity is, therefore, a therapeutic strategy used to reduce blood glucose levels, making it a crucial target for modulating postprandial hyperglycemia modulation in Type 2 diabetes mellitus [19]. Commonly used α-glucosidase inhibitors for treatment of diabetes include acarbose, miglitol, and daunorubicin. Unfortunately, these drugs are associated with adverse effects, such as hepatotoxicity and gastrointestinal symptoms [20,21]. Therefore, there is a growing interest in discovering novel plant-originated medicines with minimal adverse effects in clinical experience and relatively low costs for treating DM. Several studies have explored natural hypoglycemic drugs from various kinds of medicinal plants. For instance, Reshma et al. found that the IC_50_ of sesame seed meal methanol extract against α-glucosidase was 375 μg/mL, suggesting the potential use of discarded seeds for antidiabetic treatment [22]. Additionally, Choi et al. reported that the 75% ethanol extract from *Perilla frutescens* seed meal exhibited significant inhibition against α-glucosidase with IC_50_ values of 361.28  ±  2.01 μg/mL. This was comparable to the IC_50_ value of the positive control acarbose, which was 184.90  ±  23.23 μg/mL [23].

*Panax ginseng*, a traditional Chinese medicine and tonic, boasts a rich history of use spanning thousands of years. This revered herb has transcended its cultural origins and gained global popularity, emerging as an invaluable nutraceutical and medicinal plant [24,25]. Wild-grown ginseng, owing to overexploitation and a lengthy growth period, is almost exhausted. In response, artificially cultivated ginseng in fields has become the main product on the market, which also requires over five years to reach commercial requirements. To address these challenges, ginseng tissue cultures, specifically stem cells and adventitious roots, have emerged as an alternative approach to obtaining valuable products. They offer particular advantages, including high yield, ease of control, a shorter growth period, and the ability for easy mass production on an industrial scale [26,27]. Modern pharmacological research has indicated that ginsenosides, polysaccharides, flavonoids, and phenolics are the primary bioactive substances found in ginseng taproots, with ginsenosides standing out as the main bioactive components. Ginseng has been recognized for its various health benefits, including anti-inflammatory properties [28], central nervous system protection [29], anticancer effects [30], antidiabetic properties [26,31,32,33,34,35], cardiovascular disease prevention [36], and immunomodulation enhancement [37].

The objectives of this work were to assess and compare the in vitro efficacy of crude saponin (ginsenosides) extracts obtained from cultured cambial meristematic cells, adventitious roots, and cultivated ginseng in terms of their xanthine oxidase and α-glucosidase inhibitory activities. Additionally, this study aimed to determine the contents of total saponins, crude proteins, phenolics, and flavonoids present in the saponin extracts.

## 2. Materials and Methods

### 2.1. Chemicals, Reagents, and Different Ginseng Roots

Xanthine oxidase (EC 1.17.3.2, 128 kDa), xanthine (purity ≥ 99.5%), and vanillin were purchased from Shanghai Macklin Biochemical Company (Shanghai, China). Allopurinol (purity ≥ 98%) was purchased from Shanghai Aladdin Bio-chemical Technology Company (Shanghai, China). Acarbose, α-glucosidase, and *p*-nitrophenyl-α-D-glucopyranoside (pNPG) were obtained from Solarbio Science & Technology Co. (Beijing, China). All other solvents were of analytical grade and were purchased from Beijing Chemical Engineering Company (Beijing, China).

The field cultivated ginseng roots (CGR) were purchased from a local market in Jilin, China. The cambial meristematic cells (CMC) and adventitious ginseng roots (AGR) derived from wild ginseng were obtained from a 1000 L air-lift bubble bioreactor containing 800 L MS medium (pH 5.8) supplemented with 3 mg/L 2,4-D (2,4-dichlorophenoxyacetic acid) and 3.5% sucrose for culturing cambial meristematic cells or 4 mg/L IBA and 3% sucrose for culturing adventitious roots. The inoculum size was 1% (*w*/*v*, fresh cambial meristematic cells weight or fresh adventitious roots weight/medium volume). The airflow rate was maintained at 0.12 vvm during the period of cultivation. The temperature was maintained at 22 ± 1 °C. The cultivation was continued for 49 days for the growth of the cambial meristematic cells or adventitious ginseng roots, and we harvested the cambial meristematic cells or adventitious ginseng roots at the end of the cultivation. The purchased ginseng roots, cultured cambial meristematic cells, and adventitious roots were dried at 50 °C in a hot-air oven until a constant weight was obtained. The dried ginseng roots, cambial meristematic cells, and adventitious roots were ground by a super-micropowder mechanical grinder as samples to extract total saponins.

### 2.2. Extraction and Measurement of Total Saponins

For the extraction of total saponins, the cambial meristematic cells, adventitious roots, and cultivated ginseng roots were dried and super-micropowdered. The powders and anhydrous methanol were mixed together in a ratio of 1:20 (weight/volume) in a brown glass bottle. Then, the mixtures were put into an ultrasonic device (Kunshan, China) at 500 W for thirty minutes to extract total saponins. The obtained extracts were centrifuged at 10,000× *g* revolutions per minute for ten minutes and supernatants were collected. The same procedure was repeated twice and the supernatants were pooled together. The mixed supernatants were evaporated at 40 °C for dryness and extracted 3 times with two milliliters of water-saturated *n*-butanol. The water-saturated *n*-butanol extracts were evaporated at 60 °C for dryness. Finally, the dried matter was dissolved in distilled water and preserved at −20 °C for later use.

For the measurement of total saponins contents, the ginsenoside Re (Master Biotechnology Co., Chengdu, China) was used as a standard for standard curve establishment. Briefly, 20 µL of the total saponin extraction was mixed in 5 milliliters of 72% sulfuric acid and 0.5 milliliters of 8% vanillin ethanol in a tube. The test tube was placed in a water bath at 60 °C for ten minutes and immediately cooled in an ice water bath for ten minutes. The absorbance was measured at 544 nm wavelength with the reaction solution without samples as a blank control. The concentration of total saponins was calculated according to the standard curve.

### 2.3. Measurement of Total Phenolic, Total Flavonoid, Total Proteins

The total phenolic content was measured by the Folin–Ciocalteu assay. Briefly, 1 milliliter of saponin extracts or gallic acid standard was put into test tubes, mixed with 1 mL 0.25 mol/L Folin–Ciocalteu reagent, and left to stand for 3 min at 25 °C. Next, two milliliters of 15% Na_2_CO_3_ was added to test tubes, mixed, and kept for 15 min at 25 °C. Finally, the absorbance was measured at 760 nm. The total phenolic content was calculated according to the standard curve.

The total flavonoid content was measured by an aluminum nitrate assay. Briefly, 5 mL of saponin extract or rutin standard was put into the tubes, mixed with 0.3 milliliters of 5% NaNO_2_, and kept for 5 min at 25 °C. Next, 0.3 milliliters of 10% AL(NO_3_)_3_ was added to test tubes, mixed, and kept for 6 min at 25 °C. Then, 4 milliliters of 1 mol/L NaOH was added to the tubes and mixed. Finally, 0.4 milliliters of 30% ethanol was added and kept for ten minutes. The absorbance was measured at 510 nm. The total flavonoid content was calculated according to the standard curve.

Total proteins were determined by the Bradford method. Briefly, 1 milliliter of sample or bovine serum albumin standard was added into the tubes. Next, 5 milliliters of Bradford reagent was added to the tubes and kept for 5 min. The absorbance was measured at 595 nm. The crude protein content was calculated according to the standard curve.

### 2.4. Xanthine Oxidase Inhibitory Assay

The xanthine oxidase inhibitory ability was assayed by uric acid estimation carried out in a 96-Well Cell Culture Cluster Costar 3599 (Corning Incorporated, Corning, NY, USA) with a total volume of 200 μL containing PBS (0.10 mol/L, pH = 7.40) (140.5 μL), sample extract solution (7.5 μL) with a concentration of 0.1, 0.2, 0.4, 0.8, or 1.6 mg/mL for AGR, CMC, and CGR, and XO (10 U/mL) (2 μL). These mixtures were pre-incubated under 25 °C for 30 min, then 53.5 μL xanthine (250 mM) was added, reacted for 30 min, and finally, 6.5 μL HCl (6 M) was added to terminate the chemical reaction. The absorbance of the reaction was assayed by a microplate reader (Synergy STX, BIO-TEK, Agilent, Santa Clara, CA, USA) at a detection wavelength of 295 nm. The allopurinol at a concentration of 0.0125, 0.025, 0.05, 0.01, or 0.2 mg/mL served as the positive. The inhibition rate was calculated based on the following equation [13]:

(1) Inhibitionrate (%)=[(A−B)−(C−D)]/(A−B) × 100%

where A represents the OD at 295 nm with enzyme and no sample, B represents the OD at 295 nm with no sample and enzyme, C represents the OD at 295 nm with sample and enzyme, and D represents the OD at 295 nm with sample and no enzyme.

### 2.5. α-Glucosidase Inhibitory Measurement

In vitro α-glucosidase inhibitory abilities were carried out in a 96-Well Cell Culture Cluster Costar 3599 (Corning Incorporated) by the *p*-NPG method. Firstly, 44 μL of PBS (pH 6.8), 20 μL of sample extract solution with a concentration of 0.05, 0.1, 0.2, 0.4, or 0.8 mg/mL for AGR and CMC, and 0.0125, 0.025, 0.05, 0.01, or 0.2 mg/mL for CGR in PBS (pH 6.8) and 20 μL of α-glucosidase (10 U/mL) were mixed in a well and incubated at 37 °C for 30 min. Next, 16 μL of 40 mM para-nitrophenyl-α-d-glucopyranoside was added into the well and incubated at 37 °C for 90 min. At last, the reaction was stopped by adding 100 μL of 1 M Na_2_CO_3_. The absorbance was measured at 405 nm by a microplate reader (Synergy STX, BIO-TEK). Acarbose at a concentration of 0.0125, 0.025, 0.05, 0.01, or 0.2 mg/mL was used as the positive control. The inhibition rate was calculated based on the Equation (1), where A represents the OD at 405 nm with enzyme and no sample, B represents the OD at 405 nm with no sample and enzyme, C represents the OD at 405 nm with sample and enzyme, and D represents the OD at 405 nm with sample and no enzyme [38].

## 3. Results

### 3.1. Comparison of XO Inhibitory Activities among AGR, CMC, and CGR

**Figure 1:**
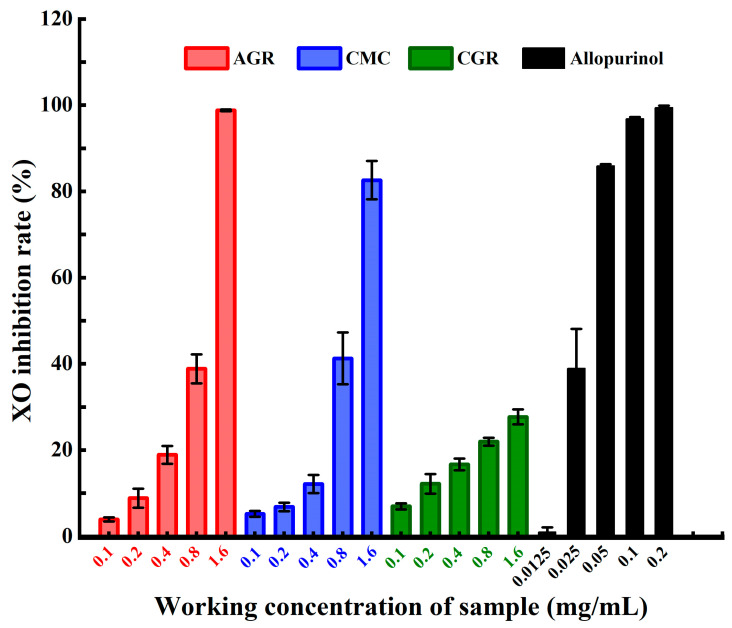
The xanthine oxidase inhibitory activities of the total saponin extracts from AGR, CMC, and CGR as well as the positive control allopurinol. Data are presented as means ± SD.

Figure 1 illustrates the results of the XO inhibitory activities of the total saponin extracts from AGR, CMC, and CGR. The XO inhibitory activities of AGR, CMC, and CGR were 3.945 ± 0.475~98.788 ± 0.201%, 5.226 ± 0.688~82.603 ± 6.29%, and 6.945 ± 0.712~27.68 ± 1.734%, respectively, at concentrations ranging from 0.1 mg/mL to 1.6 mg/mL. AGR showed the highest inhibitory activity of 98.788 ± 0.201%, followed by CMC with 82.603 ± 6.29%, at a concentration of 1.6 mg/mL. However, CGR showed the lowest inhibition rate, only 27.68 ± 1.734% at a concentration of 1.6 mg/mL. The positive control, allopurinol, demonstrated more significant inhibition activity against XO, with an inhibition rate of 96.853 ± 0.365% at a concentration of 0.1 mg/mL. In comparison, AGR, CMC, and CGR exhibited lower inhibition rates, 3.945 ± 0.475~6.945 ± 0.712% at the same concentration. The calculated IC_50_ values, representing the sample concentration that inhibited 50% of XO activity, were 0.665 mg/mL for AGR, 0.844 mg/mL for CMC, and >1.6 mg/mL for CGR. The IC_50_ value for the positive control allopurinol in this research was 0.034 mg/mL. The IC_50_ values confirmed that the total saponin extracts of adventitious ginseng roots and cambial meristematic cells against XO have more efficacy than cultivated ginseng roots.

### 3.2. Comparison of α-Glucosidase Inhibitory Activities among AGR, CMC, and CGR

**Figure 2:**
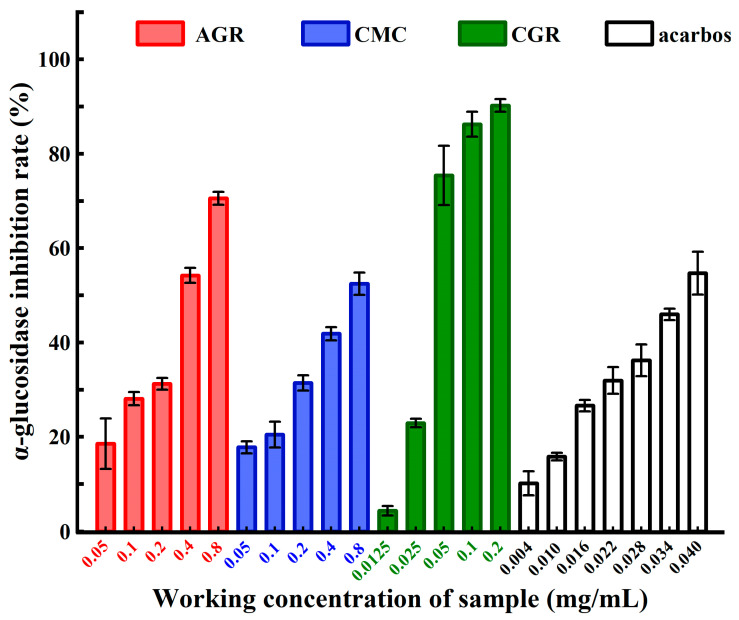
The α-glucosidase inhibitory rates of the total saponin extracts from AGR, CMC, and CGR as well as the positive control acarbose. Data are presented as means ± SD.

Figure 2 presents the results of the α-glucosidase inhibitory activities of the total saponin extracts from AGR, CMC, and CGR. The α-glucosidase inhibitory rates for AGR and CMC, at concentrations ranging from 0.05 mg/mL to 0.8 mg/mL, were 18.542 ± 5.356~70.523 ± 1.363% and 17.75 ± 1.248~52.456 ± 2.361%, respectively. Since the inhibition rate for CGR exceeded 70% at the lowest concentration of 0.05 mg/mL, the concentration range for its inhibition activity determination was adjusted to 0.0125~0.2 mg/mL. The α-glucosidase inhibition rate of CGR was 4.328 ± 1.012~90.212 ± 1.349% within this concentration range. For the positive control acarbose, the α-glucosidase inhibitory activity was 10.132 ± 2.543~54.682 ± 4.543% at concentrations from 0.004 mg/mL to 0.040 mg/mL. The IC_50_ values for AGR, CMC, CGR, and the positive control acarbose were 0.332, 0.745, 0.042, and 0.037 mg/mL respectively. The IC_50_ results suggest that CGR exhibits the highest α-glucosidase inhibitory ability, which is equivalent to the positive control acarbose, while AGR demonstrates better α-glucosidase inhibitory ability than CMC.

### 3.3. Comparison of Crude Proteins, Total Phenolics, and Flavonoids Contents among AGR, CMC, and CGR

**Figure 3:**
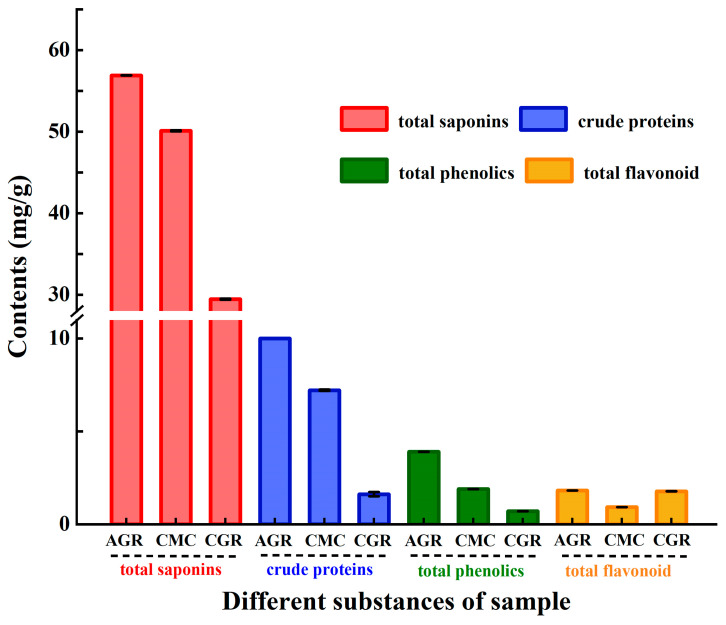
Contents of total saponins, crude proteins, total phenolics, and flavonoids among AGR, CMC, and CGR. Data are presented as means ± SD.

Figure 3 displays the contents of total saponins, crude proteins, phenolics, and flavonoids in the crude extracts of AGR, CMC, and CGR. Besides saponins, which are the primary constituents, proteins, phenolics, and flavonoids are also present in the crude extracts of AGR, CMC, and CGR. The ranking of total saponins, crude proteins, and total phenolics’ contents is AGR > CMC > CGR. The ranking of total flavonoids contents is AGR > CGR > CMC. Crude proteins and total phenolics contents in AGR and CMC are significantly higher than those in CGR, but there is no obvious difference in total flavonoids content among AGR, CMC, and CGR. In this study, the contents of total phenolics were 3.9 ± 0.001, 1.9 ± 0.001, and 0.7 ± 0.001 mg/g for AGR, CMC, and CGR, respectively. The contents of total flavonoids were 1.816 ± 0.006, 0.917 ± 0.003, and 1.775 ± 0.003 mg/g for AGR, CMC, and CGR, respectively.

## 4. Discussion

Limited research has reported on the inhibitory activity of *Panax* extracts and/or ginsenoside against XO. Specifically, extracts obtained from *Panax japlcus* roots using 60% aqueous ethanol demonstrated an inhibition rate of 41.81% against OX in vitro at a concentration of 1.0 mg/mL. In comparison, allopurinol exhibited an inhibition rate of 50.90% at the same concentration of 1.0 mg/mL [39]. Six saponins, namely ginsenoside Rd, 24(R)-majoroside R1, ginsenoside Rb2, oleanolic acid-28-O-β-D-glucopyranoside, notoginsenoside Fe, and chikusetsusaponin Iva, were isolated from the extracts. Their corresponding IC_50_ values against OX were determined to be 1.86 ± 0.15, 2.08 ± 0.18, 2.71 ± 0.17, 3.52 ± 0.21, 4.20 ± 0.26, and 5.03 ± 0.36 mg/mL. These values were higher compared to those of AGR and CMC. These results were anticipated to be useful in ascertaining OX inhibitors from *Panax japlcus* and effectively designing drugs for the prevention as well as treatment of gout [39]. Both AGR and CMC demonstrated higher inhibitory activity compared to saengmaeksan (SMS) 30% ethanol extract (SMS-E), which had an IC_50_ value of 1.221 mg/mL. Saengmaeksan is a traditional Korean medicine used for treating respiratory and cardiovascular disorders, composed of three kinds of herbs including *P. ginseng* [40]. Twenty-five ginsenoside monomers were identified from the extracts; all of them were found in higher amounts in SMS-E compared to SMS-W (water extract). In a rat model of potassium oxonate-induced hyperuricemia, SMS-E was observed to reduce xanthine oxidase activities not only in the serum but also in the liver. Based on these findings, SMS is regarded as a potential candidate therapy for the treatment of hyperuricemia and gout [40]. Total saponins extracted from *Dioscorea septemloba* (TSD) have been utilized in clinical treatment of gout in China for several years. In a study conducted by Chen et al., it was reported that serum urate levels remarkably decreased in rats treated with both low and high doses of total saponins, using a rat model. Furthermore, the total saponins demonstrated efficacy similar to that of allopurinol, a commonly used medication for managing gout [41]. In a study using hypouricemic rats, induced by high cholesterol and a high fat-diet, it was confirmed that gypenosides, natural saponins extracted from *Gynostemma pentaphyllum,* significantly decreased the concentration of serum uric acid. Additionally, the gypenosides were found to reduce the activities of xanthine oxidase (XOD), adenosine deaminase (ADA), and xanthine dehydrogenase. These results suggest that gypenosides may be effective in treating hyperuricemia by reducing xanthine oxidoreductase activity [42].

Limited research has reported on the inhibition activity of *Panax* AGR and CMC on α-glucosidase. In vivo results from the study indicated that the extract from tissue culture-raised mountain ginseng adventitious root extract (TCMGARs) at dosage amounts of 250 and 500 mg/kg body weight showed more significant lowering activities on blood glucose, total cholesterol, and triglyceride concentration in streptozotocin-induced diabetic rats compared to a field-cultivated Korean ginseng root extract. This positive efficacy might be attributed to the higher ginsenoside contents and different composition of TCMGARs [31]. The observed discrepancy in results, where field-cultured CGR showed more inhibition activity than tissue-cultured AGR on α-glucosidase, contrasts to our findings. Additionally, the study explored the effects of irradiated ginseng adventitious root saponin extract exposed to 5 kGy ^60^Co γ-rays at a dosage of 500 mg/kg·BW. This irradiated extract exhibited obviously hypoglycemic effects, with blood glucose levels in the 500 mg/kg·BW group similar to those treated with metformin and obviously lower than the group treated with the same dosage of unirradiated ginseng adventitious root saponin extract (*p* < 0.05) [26]. In the two previous cases, the hypoglycemic effects observed were not explicitly attributed to the inhibition of α-glucosidase. However, more recent research has reported the IC_50_ values of certain ginsenosides against α-glucosidase. Notable examples include ginsenoside Rg1 (IC_50_ = 5.34 mg/mL, acarbose IC_50_ = 6.54 mg/mL), ginsenoside F4 (IC_50_ = 16.97 mg/mL, acarbose IC_50_ = 3.39 mg/mL), ginsenoside Rc (IC_50_ = 39.75 mg/mL, acarbose IC_50_ = 3.39 mg/mL) [38,43]. The inhibition rates against α-glucosidase of ginsenoside Rg6, ginsenoside Ro, ginsenoside Ra1, and ginsenoside Ra2 at a concentration of 40 mM (30.68~48.46 mg/mL) were found to be 27.35%, 20.23%, 16.36%, and 29.54%, respectively [43]. All these ginsenoside monomers showed significantly lower inhibition activities compared to AGR and CMC total saponin extracts. In a study by Wang et al., the inhibitory activity against α-amylase of the extracts from the white ginseng and red ginseng was compared in vitro. Results showed that white ginseng extracts (IC_50_ was 20.10 ± 3.48 mg/mL) exhibited a higher inhibition ability than that of the red ginseng extracts (IC_50_ was 48.38 ± 1.02 mg/mL). The decrease in inhibition ability against α-amylase in red ginseng was due to the obvious reduction in the amount of total saponins. Further, eight ginsenosides (Rb1, Rb3, Rc, Rd, Rg2, Rh1, Rb2, and Rf) were found to inhibit α-amylase, with IC_50_ values ranging from 0.034 ± 0.001 mg/mL to 0.202 ± 0.009 mg/mL. Ginsenoside Rb1 (IC_50_ was 0.034 ± 0.001 mg/mL) presented the greatest inhibition ability against α-amylase, similar to the positive control acarbose (IC_50_ was 0.033 ± 0.002 mg/mL), while ginsenoside Rf (IC_50_ was 0.202 ± 0.009 mg/mL) showed the lowest inhibition ability against α-amylase [44]. Interestingly, some researchers compared the anti-diabetic efficacy of protopanaxadiol-type saponins and protopanaxatriol-type saponins in animals with type 1 diabetes (T1D) or type 2 diabetes mellitus (T2DM). Results from Ju et al., found that the diol-ginsenoside fraction, as opposed to the triol-ginsenoside fraction extracted from Korean red ginseng, delayed the onset and reduced the incidence of type 1 diabetes (T1D) in diabetes-prone biobreeding rats [32]. On the other hand, Deng et al. reported that both protopanaxadiol- and protopanaxatriol-type saponins, at low doses (50 mg/kg·BW) or high doses (150 mg/kg·BW), significantly decreased fasting blood glucose and improved glucose tolerance and insulin resistance in high-fat diet/streptozocin-induced T2DM mice. This suggests that both protopanaxadiol- and protopanaxatriol-type saponins may act as natural anti-diabetic substances to be used for prevention and treatment for T2DM and its complications in the future [45]. Currently, approximately two hundred ginsenoside monomers have been identified from ginseng plants and their heat-processed products [35]. Future research should focus on determining which individual ginsenosides possess the highest inhibition activity against α-glucosidase in AGR and CMC total saponin extracts as well as exploring potential synergistic actions among them.

The contents of total phenolics and total flavonoids in the present study were found to be lower than those reported by Kim [46] and Malathy et al. [47]. Malathy et al. specifically reported that the contents of total phenolics and flavonoids in the methanol extract of ginseng roots were 30.21 mg/g and 20.25 mg/g, respectively. Discrepancies in these values may arise from extraction conditions such as higher extraction temperature and/or longer extraction time employed in the referenced studies [46,47]. Additionally, differences in ginseng varieties used as source materials could also contribute to the observed variations.

While there is no specific report on the inhibitory activity of proteins, phenolics, and flavonoids from ginseng on xanthine oxidase, studies on bioactive peptides from other food sources have demonstrated xanthine oxidase inhibitory activity. For instance, three food-derived bioactive peptides—VW4 from the hydrolysates of *Scomber austriasicus* meat by the enzyme of papain, VS14 from tuna-backbone protein digested by pepsin, and IW3 from the ovotransferrin hydrolysates of egg white by thermolysin-pepsin—have shown xanthine oxidase inhibitory activity with IC_50_ values of 3.611 ± 0.105, 2.740 ± 0.022, and 1.338 ± 0.015 mg/mL respectively (compared to the IC_50_ of allopurinol, 0.171 ± 0.001 mg/mL). This suggests that these three peptides could potentially serve as natural XOD inhibitors [48]. Additionally, results from studies on medicinal plants suggest that phenolics and flavonoids have significant potential as new xanthine oxidase inhibitors capable of use against gout disease [49].

Ginseng saponins, known as ginsenosides, are regarded as bioactive substances responsible for the anti-diabetic effect [50]. Yet, phenolics and flavonoids from other plants, such as *Sorghum bicolor*, *Musella lasiocarpa*, and *Euphorbia resinifera* [51,52,53], have shown inhibitory activities against α-glucosidase and xanthine oxidase. For example, phenolic extracts from raw red sorghum grains (*Sorghum bicolor*) exhibited an IC_50_ value of 10.78 ± 0.63 μg/mL against α-glucosidase (acarbose, 18.04 ± 1.25) and 28.35 ± 1.86 µg/mL against XO (allopurinol, 7.04 ± 0.44 µg/mL) [51]. The crude methanol extract from flowers of *Musella lasiocarpa* showed the highest inhibition activity against xanthine oxidase (IC_50_, 5.23 ± 0.35 µg/mL) compared to allopurinol (IC_50_, 24.85 µg/mL). However, it showed an IC_50_ of 79.15 ± 6.03 μg/mL against α-glucosidase (acarbose IC_50_, 125.66 ± 6.13 µg/mL). The EtOAc fraction exhibited the best inhibition activity against α-glucosidase with an IC_50_ value of 18.86 ± 0.44 µg/mL but showed an IC_50_ of 7.50 ± 0.16 µg/mL against xanthine oxidase. This fraction contained phenolic acids, fatty acids, esters, terpenoids, and flavonoids [52]. Water and ethanol extracts from the aerial part of *E. resinifera* containing total phenols and flavonoids showed inhibition against α-glucosidase with IC_50_ values of 121.4 ± 1.88 µg/mL and 56.6 ± 1.12 µg/mL (acarbose, 18.01 ± 2.00 µg/mL) and inhibition against xanthine oxidase with IC_50_ values of 69.83 ± 1 µg/mL and 10.26 ± 0.6 µg/mL (allopurinol, 0.78 ± 0.01 µg/mL), respectively. These results suggest that phenolics and flavonoids from plants have more inhibitory activity against α-glucosidase and/or xanthine oxidase. Whether ginseng saponins as well as crude proteins, total phenolics, and flavonoids in ginseng extracts act individually or synergistically against XO and α-glucosidase needs further elucidation.

AGR and CMC can be produced on an industrial scale under controlled culture conditions. Their crude saponin (ginsenoside) extracts, containing varying amounts of saponins, crude proteins, phenolics, and flavonoids, have shown higher inhibitory activities against xanthine oxidase and α-glucosidase. This suggests the importance of further elucidating the individual compounds within these extracts and understanding their specific biological activities. Such investigations can contribute to drug discovery and promoting healthy food production.

## 5. Conclusions

The total saponin extracts from AGR and CMC exhibited higher inhibitory activities against xanthine oxidase compared to CGR. However, their inhibitory activities on α-glucosidase were lower than that of CGR. The extracts of AGR and CMC also had higher contents of total saponins, crude proteins, and total phenolics compared to CGR. Moreover, the total flavonoids content in AGR extracts was higher than that in CGR, while it was lower in CMC extracts compared to CGR. The results suggest that total saponin extracts from AGR and CMC hold potential for the development of medicinal, nutraceutical, and functional products with dual benefits of hypoglycemic and antihyperuricemic effects.

## Data Availability

The data presented in this study are available on request from the corresponding author.
